# From Obesity Resistance to Obesity Prediction and Prevention?

**DOI:** 10.3389/fnins.2016.00369

**Published:** 2016-08-09

**Authors:** Silvana Gaetani, Tommaso Cassano

**Affiliations:** ^1^Department of Physiology and Pharmacology “V. Erspamer,” Sapienza University of RomeRome, Italy; ^2^Department of Clinical and Experimental Medicine, University of FoggiaFoggia, Italy

**Keywords:** diet induced obesity, hypothalamus, neuropeptides, gene expression, DNA methylation, high fat diet

Eating disorders and obesity are urgent public health problem with a high burden of diseases for our societies. Core symptoms are unhealthy eating habits, with disturbances of eating behavior, and inability to control body weight.

Food intake and energy balance are controlled as a complex, redundant, regulatory system ensuring that the amount of ingested calories does not exceed what the body can safely handle and that sufficient body fuel is stored as fat. The main brain regions integrating peripheral and central signals of this system are the hypothalamus and the brainstem. Key hypothalamic neurons are those co-expressing neuropeptide Y (NPY) and agouti-related protein (AgRP), which stimulate eating, and those expressing pro-opiomelanocortin (POMC) and cocain- and amphetamine-regulated transcript (CART), which inhibit feeding (Barsh and Schwartz, 2002). These mechanisms have not changed in the last 30 years (Richard, 2015), while dramatic changes occurred in the sensory side of the control process. Increased palatability, variety, availability, and energy content of food created an “obesogenic food environment” able to overwhelm the body regulatory system and cause an “obesity epidemic” (Johnson and Wardle, 2014; Lipek et al., 2015).

Differences in the responsiveness to “external” food cues and “internal” homeostatic signals might be at the crossroad between obesity resistance and obesity development, although the mechanisms at the basis of these differences are not fully elucidated (Llewellyn and Wardle, 2015).

It is well-known that the regulation of chromatin structure can modulate the accessibility of chromatin to transcription factors, thus regulating gene expression (Russo et al., 1996). This is the core mechanism of the epigenetic control, through which environmental factors are able to modulate the expression of the genotype into the phenotype influencing the progression of different diseases, including eating disorders (Pucci et al., 2015 Addiction Biology). Silencing and unsilencing of genes can occur via changes in DNA methylation, as well as through epigenetic modifications at the level of the histones (Feng et al., 2007). Nutritional epigenomics have suggested that in most cases the development of obesity and type2 diabetes might be attributed to stable epigenetic alterations occurring in early nutrition, during gestation and lactation (Gallou-Kabani and Junien, 2005). Whether similar mechanisms take place also in adulthood, following the exposure to an “obesogenic” environment and whether they are involved in obesity development remained less investigated.

This hypothesis was the aim of a recent study in an animal model of diet-induced-obesity (DIO) by two Italian research teams, led by Carlo Cifani and Claudio D'Addario (Cifani et al., 2015). The authors investigated the individual sensitivity to weight gain/resistance of adult male Sprague Dawley rats, exposed to a fat-enriched (45%) hypercaloric (5.24 kcal/g) diet and evaluated the hypothalamic gene expression and the epigenetic regulation of NPY, AgRP, POMC, CART, and of two receptors, namely PPAR-γ and leptin receptor (lepR). The authors focused on two different time-points with respect to the exposure to a high-fat diet (HFD). At the first one (5 weeks) two subsets of HFD-fed rats started showing divergent body weight gains: One subset was slightly, but significantly, heavier than control rats (receiving a standard laboratory chow of 2.6 kcal/g), indicating the development of the DIO phenotype, while the other subset maintained the same body weight gain of control rats, showing an obesity-resistant (DR) phenotype. At the second time point of 21 weeks these two phenotypes were utmost evident, with DIO rats weighting 21% more than DR rats. Orexygenic NPY and PPAR-γ genes resulted to be up-regulated in DIO rats with respect to DR rats after 5 weeks of HFD exposure, but not at the latest time-point, and a consistent increase in DNA methylation at specific sites of the gene promoter was also observed for NPY. Conversely, POMC expression was down-regulated in DIO rats when obesity was present from several weeks and, also in this case, the authors found a consistent change of DNA methylation at the gene promoter for POMC. The interesting hypothetical interpretation that the authors offer is that NPY and PPAR-γ might be involved in the early events leading to the onset of the obese phenotype, but not later, when obesity is established and likely maintained by the reduction of POMC-derived signals, such, as, for example melanocortins. In keeping with their hypothesis, the epigenetic regulation of these gene expressions seems to be transient and reversible. This finding is particularly new and open novel perspectives in the attempt of dissecting the mechanisms responsible for obesity development in modern societies.

Future studies should broaden these results, taking into consideration also other possible targets in the hypothalamus, as well as in other brain areas, and involved not only in the homeostatic but also in the hedonic/emotional and psychiatric aspects of eating. For example, a good set of candidate genes might include oxytocin and oxytocin receptors, based on the key role that the oxytocinergic system plays in the metabolic/behavioral regulation of feeding and body weight as well as in the social/emotional aspects that characterize obesity and eating disorders (Romano et al., 2016). Another possible target to be evaluated is the endocannabinoid/acylethanolamide system for the accumulating evidence on its function in the modulation of satiety, anxiety, mood tone and the possible implication in “food addiction” (D'Addario et al., 2014; Romano et al., 2014, 2015).

Our increasing understanding of the elegant interconnected redundancy of the pathways governing energy homeostasis is clearly pointing at the emerging need of influencing more than one element of the system jointly to achieve effective and safe weight reduction. The pharmacological manipulation of epigenetic mechanisms might offer a unique opportunity to target at once multiple marks, thus representing a promising tool to achieve both obesity prevention and therapy. The idea is basically that if it is possible to detect early changes in the expression regulation of candidate genes via epigenetic mechanisms, it would be possible to identify biomarkers and to slow-down or even avoid the development of the disease by contrasting such epigenetic modifications (Choi and Friso, 2010). In the context of Cifani et al. paper, where it was observed an increase in DNA methylation, it could be possible to suggest the use of bioactive compounds able to inhibit DNA methylation, such as genistein, found in many food products containing soy, or other demethylating agents such as antisense DNA methyltransferase (Choi and Friso, 2010).

Besides DNA methylation, other epigenetic alterations might include histone acetylation, one of the most extensively studied histone modifications controlled by the balance between locally recruited complexes containing histone acetyl transferases (HATs) and histone deacetylases (HDACs). Imbalance of the HAT-HDAC equilibrium has been linked to a number of diseases including cancer and obesity. The events causing the HAT-HDAC imbalance are well-understood and have provided a rationale for the development of HDAC inhibitors for pharmacotherapies. Recent interest in HDAC inhibitors has expanded into dietary compounds such as butyrate, a short-chain fatty acid from fiber, diallyl disulfide, an organosulfur compound from garlic, and sulforaphane, an isothiocyanate found in cruciferous vegetables, exhibiting HDAC inhibitory activity (Choi and Friso, 2010).

In summary, if obesity development is mostly linked to wrong eating habits, oversize food portions and wrong dietary choices, we should take the opportunity to dissect the molecular mechanisms at the basis of obesity resistance in the same “obesogenic environment” both to redirect eating habits toward healthy and functional foods that can prevent obesity, both to develop novel pharmacological tools able to correct the altered mechanisms and combat obesity.

## Author contributions

All authors listed, have made substantial, direct, and intellectual contribution to the work, and approved it for publication.

### Conflict of interest statement

The authors declare that the research was conducted in the absence of any commercial or financial relationships that could be construed as a potential conflict of interest.

## References

[B1] Barsh G. S. Schwartz M. W. (2002). Genetic approaches to studying energy balance: perception and integration. Nat. Rev. Genet. 3, 589–600. 10.1038/nrg862 12154382

[B2] Choi S. W. Friso S. (2010). Epigenetics: a new bridge between nutrition and health. Adv. Nutr. 1, 8–16. 10.3945/an.110.1004 22043447 PMC3042783

[B3] Cifani C. Micioni Di Bonaventura M. V. Pucci M. Giusepponi M. E. Romano A. Di Francesco A.. (2015). Regulation of hypothalamic neuropeptides gene expression in diet induced obesity resistant rats: possible targets for obesity prediction? Front. Neurosci. 9:187. 10.3389/fnins.2015.00187 26106286 PMC4458694

[B4] D'Addario C. Micioni Di Bonaventura M. V. Pucci M. Romano A. Gaetani S. Ciccocioppo R.. (2014). Endocannabinoid signaling and food addiction. Neurosci. Biobehav. Rev. 47, 203–224. 10.1016/j.neubiorev.2014.08.008 25173635

[B5] Feng J. Fouse S. Fan G. (2007). Epigenetic regulation of neural gene expression and neuronal function. Pediatr. Res. 61, 58R–63R. 10.1203/pdr.0b013e3180457635 17413844

[B6] Gallou-Kabani C. Junien C. (2005). Nutritional epigenomics of metabolic syndrome: new perspective against the epidemic. Diabetes 54, 1899–1906. 10.2337/diabetes.54.7.1899 15983188

[B7] Johnson F. Wardle J. (2014). Variety, palatability, and obesity. Adv. Nutr. 5, 851–859. 10.3945/an.114.007120 25398751 PMC4224225

[B8] Lipek T. Igel U. Gausche R. Kiess W. Grande G. (2015). Obesogenic environments: environmental approaches to obesity prevention. J. Pediatr. Endocrinol. Metab. 28, 485–495. 10.1515/jpem-2015-0127 25928754

[B9] Llewellyn C. Wardle J. (2015). Behavioral susceptibility to obesity: gene-environment interplay in the development of weight. Physiol. Behav. 152, 494–501. 10.1016/j.physbeh.2015.07.006 26166156

[B10] Pucci M. Micioni Di Bonaventura M. V. Giusepponi M. E. Romano A. Filaferro M. MacCarrone M.. (2015). Epigenetic regulation of nociceptin/orphanin FQ and corticotropin-releasing factor system genes in frustration stress-induced binge-like palatable food consumption. Addict Biol. 10.1111/adb.12303. [Epub ahead of print]. 26387568

[B11] Richard D. (2015). Cognitive and autonomic determinants of energy homeostasis in obesity. Nat. Rev. Endocrinol. 11, 489–501. 10.1038/nrendo.2015.103 26122319

[B12] Romano A. Karimian Azari E. Tempesta B. Mansouri A. Micioni Di Bonaventura M. V. Ramachandran T. A.. (2014). High dietary fat intake influences the activation of specific hindbrain and hypothalamic nuclei by the satiety factor oleoylethanolamide. Physiol. Behav. 136, 55–62. 10.1016/j.physbeh.2014.04.039 24802360

[B13] Romano A. Tempesta B. Micioni Di Bonaventura M. V. Gaetani S. (2016). From autism to eating disorders and more: the role of oxytocin in neuropsychiatric disorders. Front. Neurosci. 9:497. 10.3389/fnins.2015.00497 26793046 PMC4709851

[B14] Romano A. Tempesta B. Provensi G. Passani M. B. Gaetani S. (2015). Central mechanisms mediating the hypophagic effects of oleoylethanolamide and N-acylphosphatidylethanolamines: different lipid signals? Front. Pharmacol. 26:137 10.3389/fphar.2015.00137 PMC4481858 26167152

[B15] Russo V. E. A. Martienssen R. A. Riggs A. D. (1996). Epigenetic Mechanisms of Gene Regulation. Plainview, NY: Cold Spring Harbor Laboratory Press.

